# Supplementary material for: The thylakoid lumen Deg1 protease affects non‐photochemical quenching via the levels of violaxanthin de‐epoxidase and PsbS
Source: Plant J. 2025 Feb 24;121(4):e17263. doi: 10.1111/tpj.17263 (PMC11848705; doi:10.1111/tpj.17263)
Supplement: Supplementary file 1 — Figure S1. Imaging PAM measurements of PSII maximum quantum yield (F v/F m). Figure S2. Light response curves of photosynthetic electron transport rates (ETRs). Figure S3. Pairwise Pearson's correlation analysis of WT and deg mutant plants under NL conditions. Figure S4. Principal component analysis (PCA) of the proteomic data of WT and deg mutant plants subjected to NL and HL conditions. Figure S5. Relative expression of NPQ1 and NPQ4 during high light exposure in WT and deg mutants. Figure S6. Differential expression of altered proteins in the deg1 mutant and WT in response to cycloheximide treatment. Table S1. Raw LFQ proteomic data of WT and deg mutants under NL, HL and recovery. Table S2. Analyzed proteomic data of WT and deg mutants under NL, HL and recovery. Table S3. Proportion of chloroplast versus cellular proteins. Table S4. Proteomic raw data and analysis of WT and deg1 with or without cycloheximide treatment. [file TPJ-121-0-s001.zip › Supp. Figures + Supp. Tab. 3_18.11.24.docx]

**
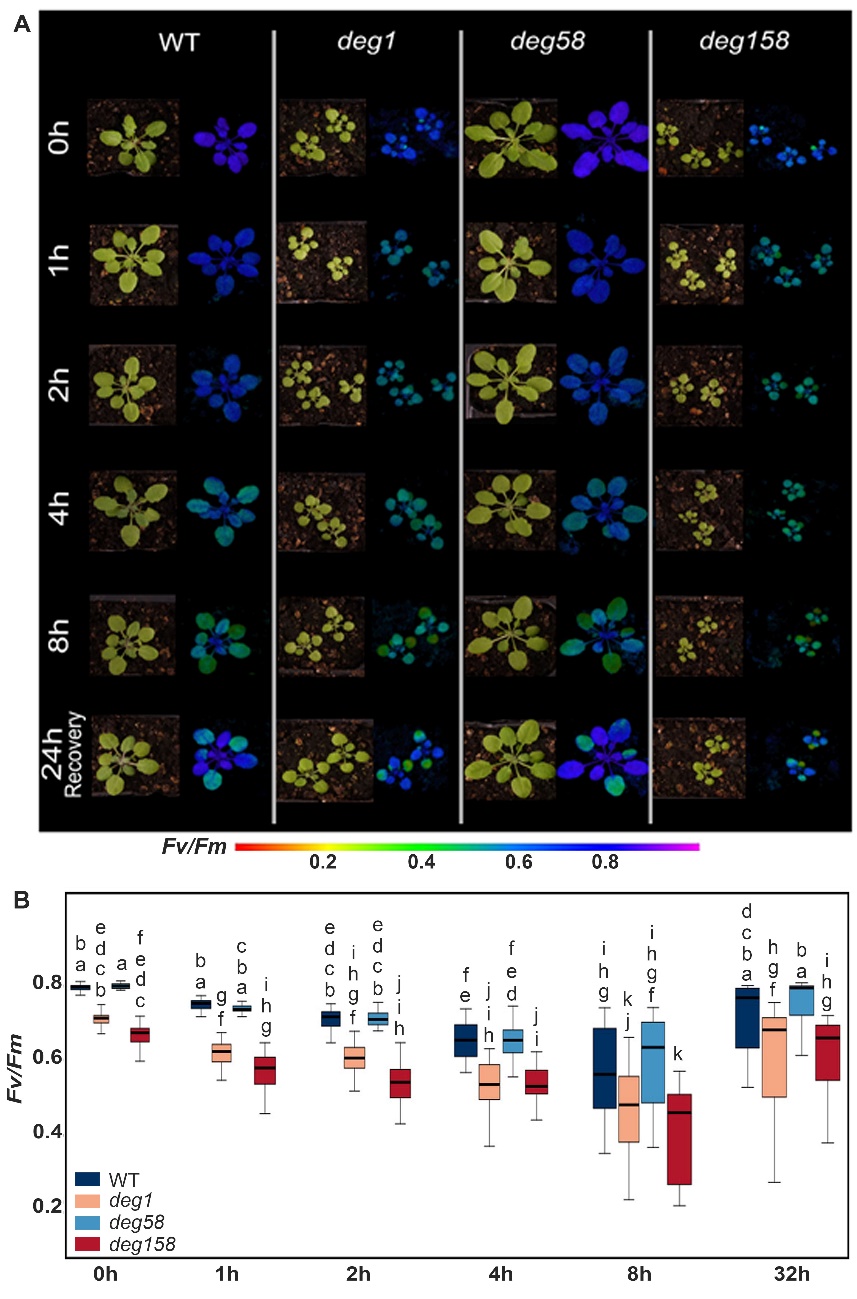
**

**Supp. Fig 1. Imaging PAM measurements of PSII maximum quantum yield (*Fv/Fm*).** (A) Images of WT, *deg1*, *deg58* and *deg158* plants and corresponding color-coded representation of *Fv/Fm* values recorded prior to (0 h) and following exposure to high-light (HL; ~750 μmol photons m^-2^ s^-1^) treatment for 1, 2, 4, and 8 h, as well as following recovery at normal light (NL) for 24 h. (B) Boxplot of *Fv/Fm* values recorded as described above for 25 leaves from at least 3 different plants, at each time point, for each genotype. Different letters indicate significant differences determined by ANOVA followed by Tukey’s test (**p*<0.05).


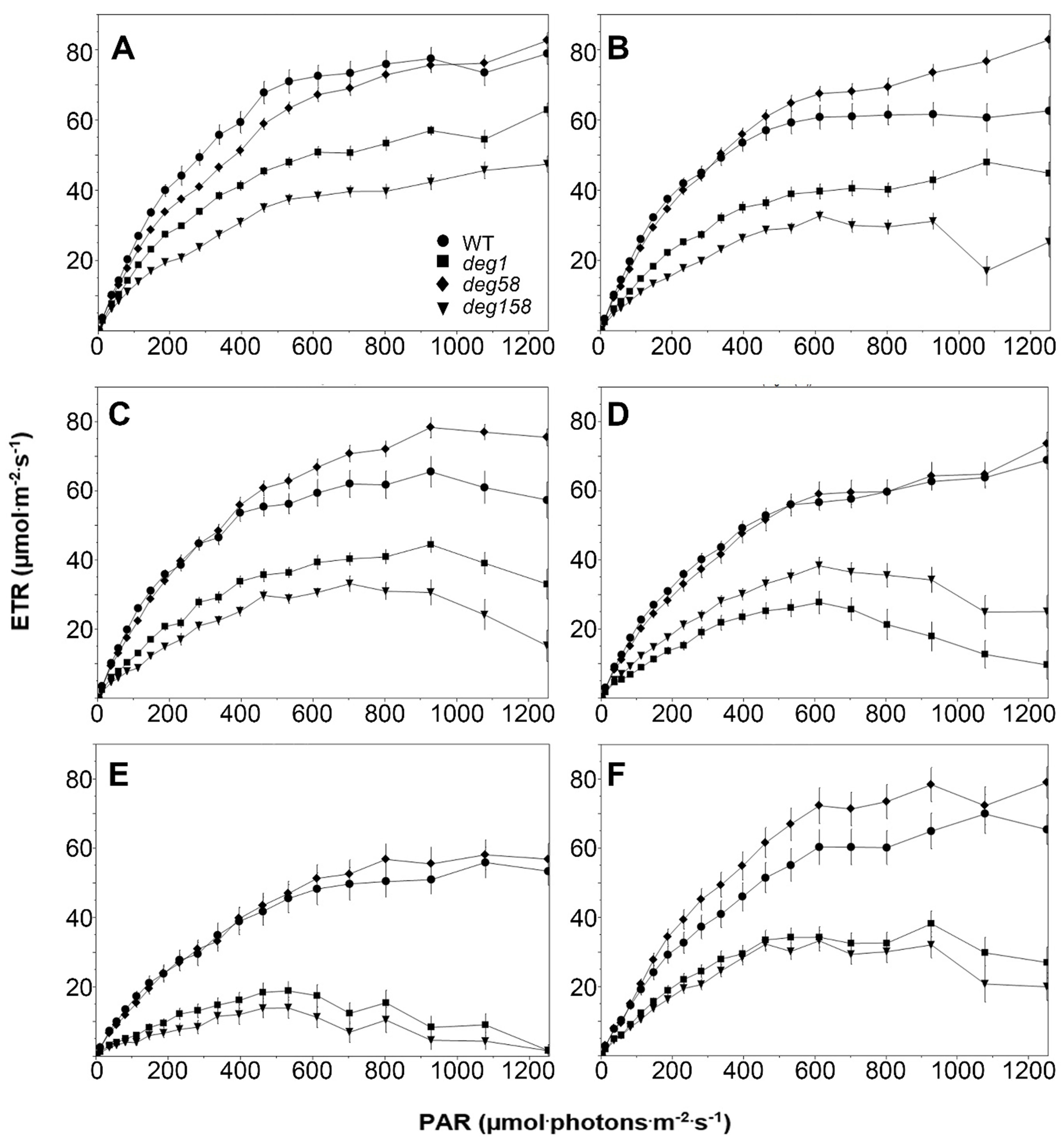


**Supp. Fig 2. Light response curves of photosynthetic electron transport rates (ETRs).** Rates were measured on intact leaves before (A) and following exposure to high-light (HL) (~750 μmol photons m^-2^ s^-1^) for 1 (B), 2 (C), 4 (D) and 8 (E) hours. (F) Rates measured on leaves exposed to HL for 8 h followed by a recovery period of 24 h under NL conditions. Values shown represent the mean ±SE obtained from recordings of 25 leaves from at least 3 different plants, at each time point, for each genotype.

| **Supp. Table 3. Proportion of chloroplast vs cellular proteins** | | |
| --- | --- | --- |
| **Genotype** | **^†^ RBCL / Actin** | **PsaA / Actin** |
| **WT** | 3698.38 ± 520.31 | 172.4 ± 15.57 |
| ***deg1*** | 4196.65 ± 526.12 | 192.58 ± 45.61 |
| ***deg58*** | 3713.15 ± 1022.2 | 190.62 ± 41.41 |
| ***deg158*** | 4279.85 ± 1083.74 | 181.36 ± 37.38 |

**^†^** Values shown are means ± SD of the ratios between LFQ values of the noted proteins at time 0. RBCL, Rubisco large subunit; PsaA, subunit A of photosystem I. No significant differences were found between the genotypes, tested by One-way ANOVA followed by post-hoc Tukey HSD Test.


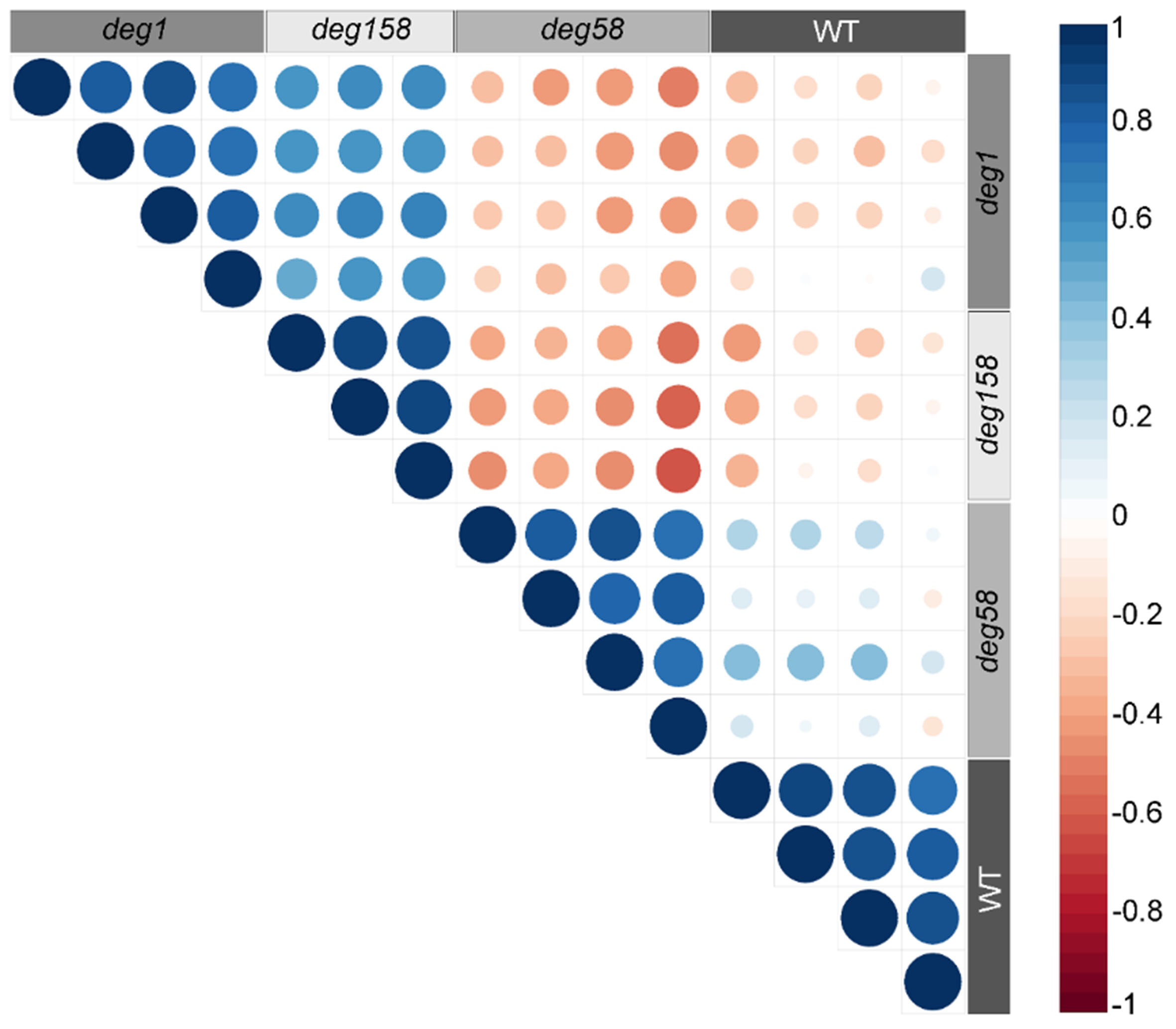


**Supp. Fig. 3. Pairwise Pearson’s correlation analysis of WT and *deg* mutant plants under NL conditions.** The triangle displays the Pearson’s correlation coefficient (*r*) between WT, *deg1*, *deg58* and *deg158* differentially expressed proteins under normal-light (NL) conditions. Blue and red indicate positive and negative correlation, respectively. The color saturation and the size of the circles reflect the magnitude of correlation. Correlation coefficient (*r*) with *p* values above 0.05 were regarded as insignificant and are colored white. The bar on the right side of the correlogram shows the Pearson correlation coefficients with their corresponding colors.

**
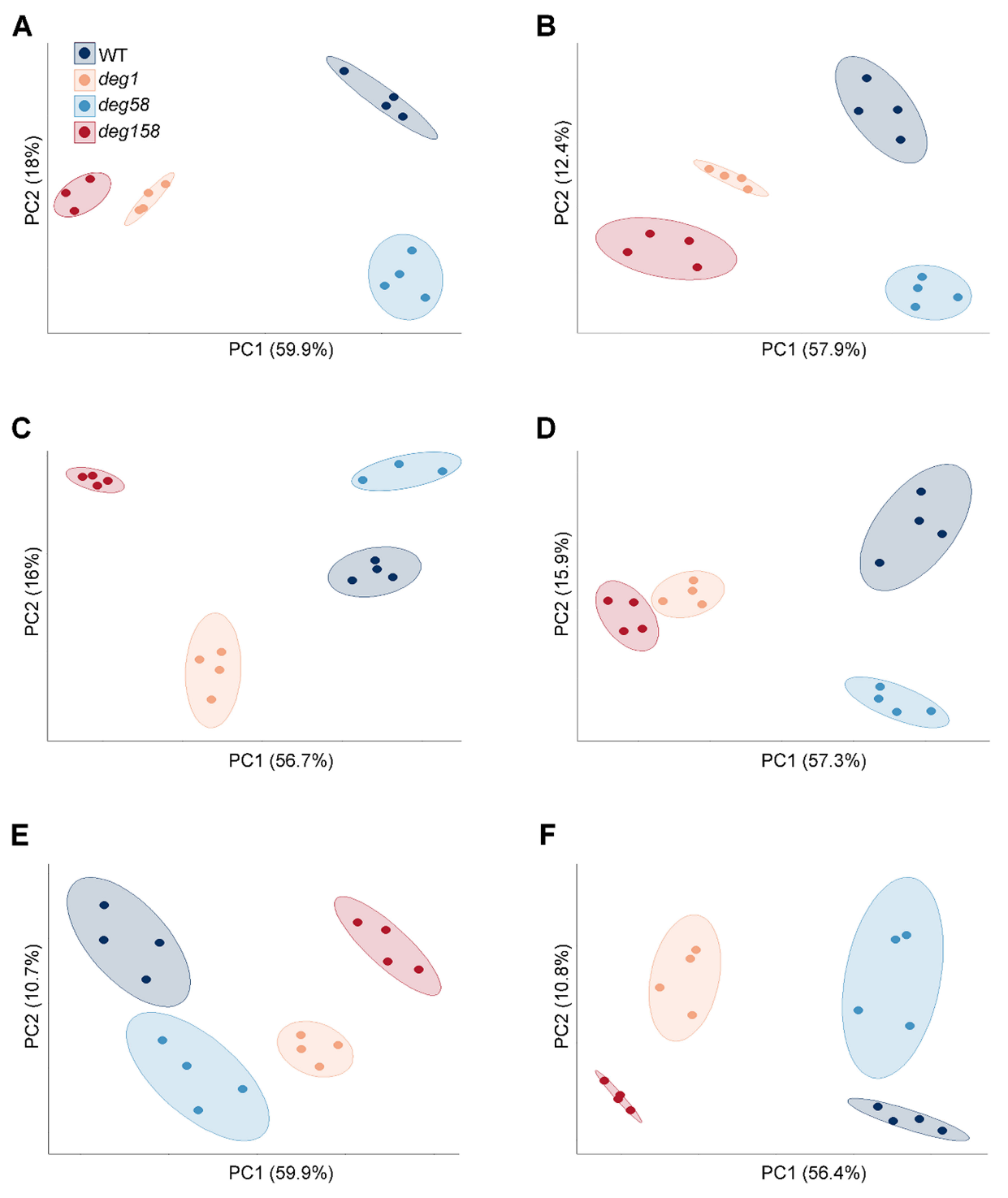
**

**Supp. Fig. 4.** **Principal component analysis (PCA) of the proteomic data of WT and *deg* mutant plants subjected to NL and HL conditions.** (A) Plants subjected to normal light (NL). (B-E) Plants exposed to high light (HL) for 1 (B), 2 (C), 4 (D), and 8 (E) hours. (F) Plants exposed to HL for 8 h followed by recovery under NL conditions for 24 h. Under all light conditions, the PCA score clearly differentiates between WT (dark blue), *deg1* (orange), *deg58* (light blue) and *deg158* (red) samples.

**
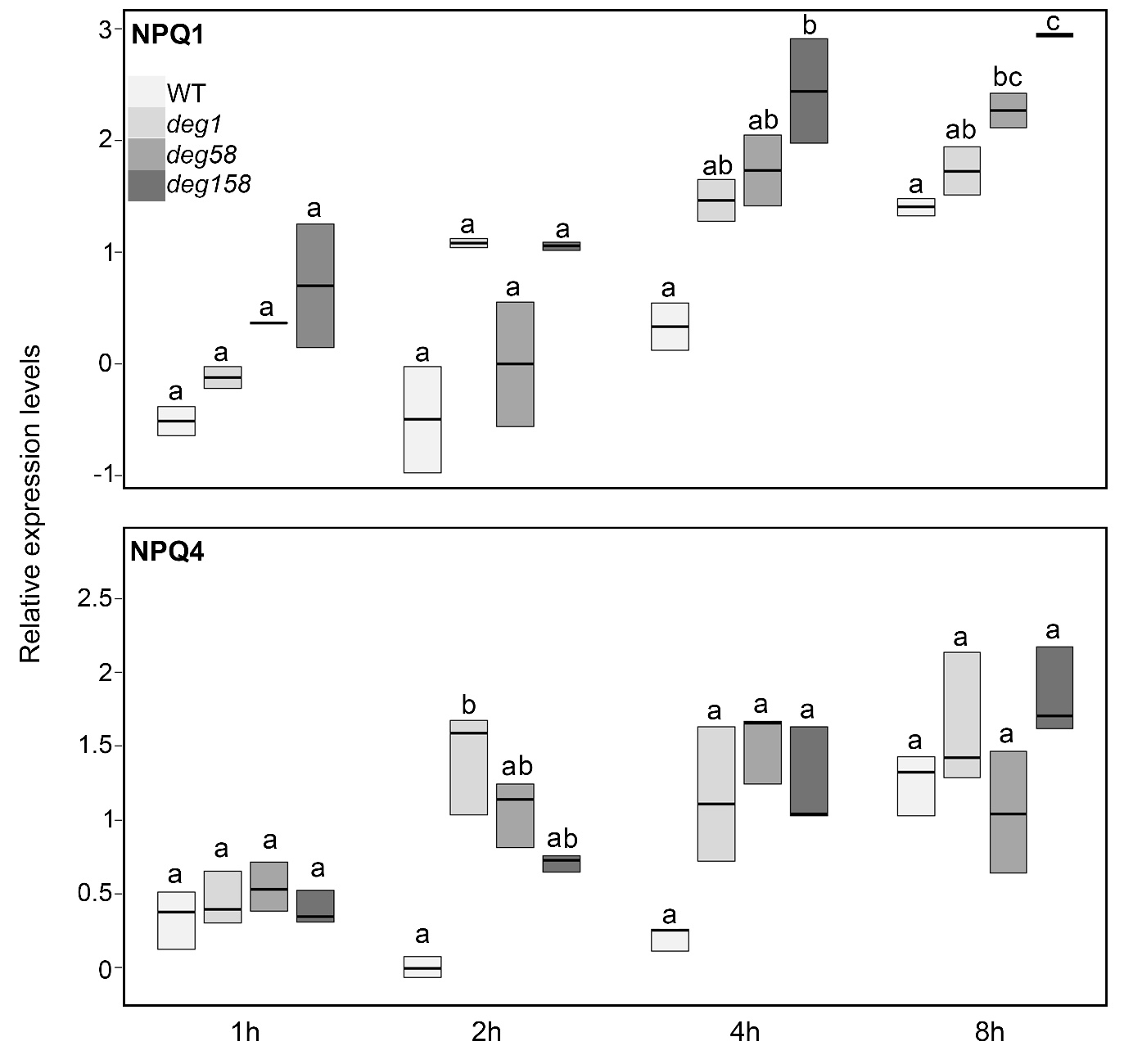
**

**Supp. Fig. 5. Relative expression of NPQ1 and NPQ4 during high light exposure in WT and *deg* mutants.** The boxplots show the relative transcript levels (i.e., log2 ratios) in WT and mutant lines 1, 2, 4 and 8 hrs after exposure to high light versus those of WT plants before high light. Different letters indicate significant differences determined by ANOVA followed by Tukey’s test (**p*<0.05).


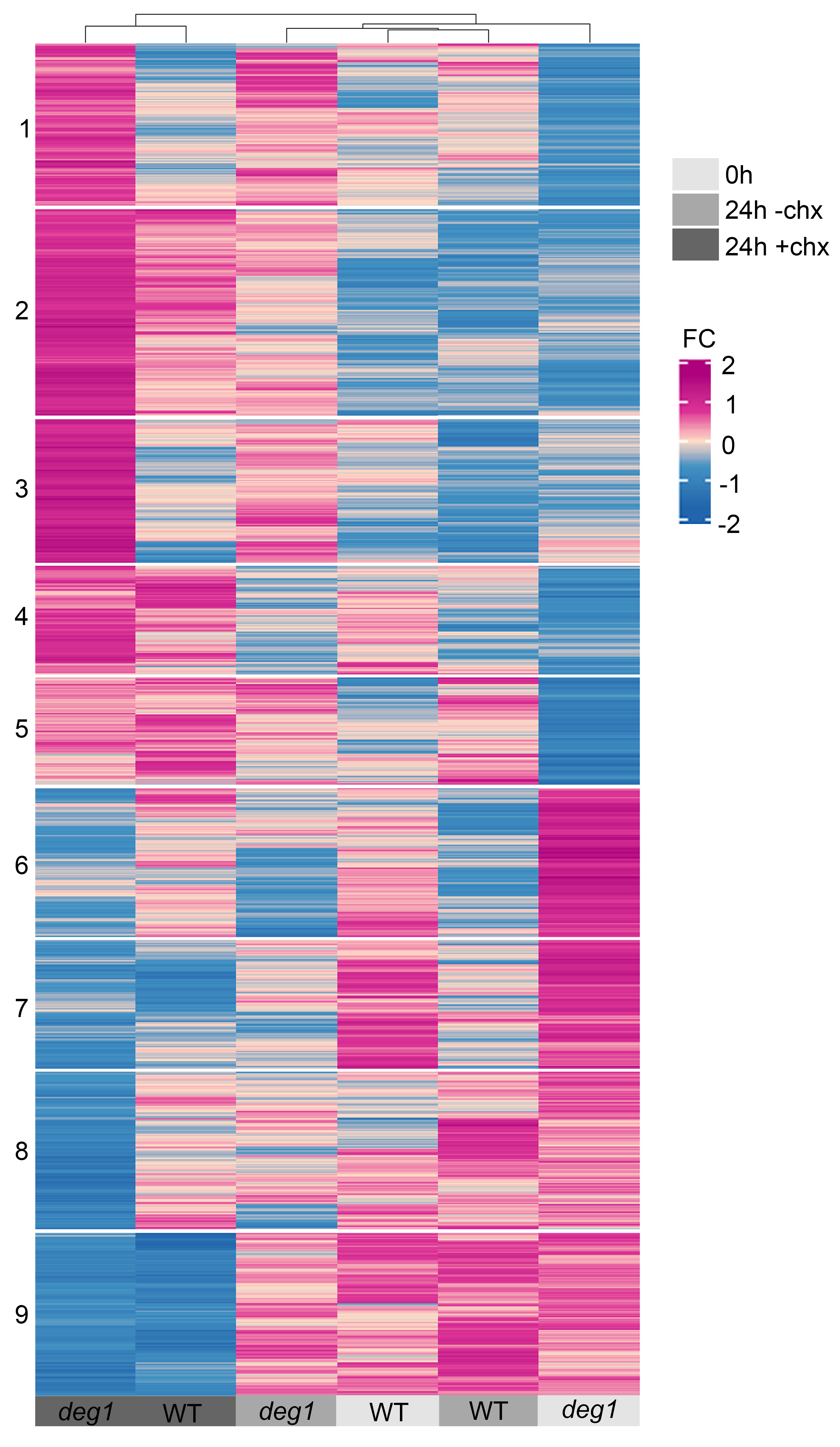


**Supp. Fig. 6. Differential expression of altered proteins in the *deg1* mutant and WT in response to cycloheximide treatment.** The heatmap was generated for the differentially expressed proteins (DEPs) in *deg1* before and following 24 h incubation with or without cycloheximide, some of which were also differentially expressed in WT. The analysis was based on the average of five replicates under different growth conditions, and reveals segregation of the 1,053 DEPs (*deg1*) into nine co-expressed groups, and mapping of both NPQ1 and NPQ4 into cluster number three (see Supp. Table 4, ‘Analyzed proteins’ – columns BB-BC).
